# Analysis of an SEIR Epidemic Model with Saturated Incidence and Saturated Treatment Function

**DOI:** 10.1155/2014/910421

**Published:** 2014-08-18

**Authors:** Jinhong Zhang, Jianwen Jia, Xinyu Song

**Affiliations:** ^1^School of Mathematics and Computer Science, Shanxi Normal University, Linfen, Shanxi 041004, China; ^2^Department of Mathematics, Xinyang Normal University, Xinyang, Henan 464000, China

## Abstract

The dynamics of SEIR epidemic model with saturated incidence rate and saturated treatment function are explored in this paper. The basic reproduction number that determines disease extinction and disease survival is given. The existing threshold conditions of all kinds of the equilibrium points are obtained. Sufficient conditions are established for the existence of backward bifurcation. The local asymptotical stability of equilibrium is verified by analyzing the eigenvalues and using the Routh-Hurwitz criterion. We also discuss the global asymptotical stability of the endemic equilibrium by autonomous convergence theorem. The study indicates that we should improve the efficiency and enlarge the capacity of the treatment to control the spread of disease. Numerical simulations are presented to support and complement the theoretical findings.

## 1. Introduction

In recent years, various epidemic models have been proposed and explored to prevent and control the spread of the infectious diseases, such as measles, tuberculosis, and flu (see e.g., [1, 2]). In many epidemic models, bilinear incidence rate *βSI* is frequently used [1, 3]. Esteva and Matias [4] introduced the saturated incidence rate *βSI*/(1 + *αI*), which tends to a saturation level when *I* gets large, *βI* measures the infection force when the disease is entering a fully susceptible population, and 1/(1 + *αI*) measures the inhibition effect from the behavioral change of susceptible individuals when their number increases or from the crowding effect of the infective individuals. This incidence rate is more reasonable than the bilinear incidence rate because it includes the behavioral change and crowding effect of the infective individuals and prevents the unboundedness of the contact rate by choosing suitable parameters. It was used in many epidemic models afterwards (see, e.g., [4, 5]).

It is well known that treatment is an important and effective method to prevent and control the spread of various infectious diseases. In classical epidemic models, the treatment rate of the infection is assumed to be proportional to the number of the infective individuals, but in general, the recovery rate depends on the medical resources, such as drugs, vaccines, hospital beds, isolation places, and efficiency of the treatment. Noting that every community or country has limited capacity for the treatment of a disease, therefore, it is very important to adopt a suitable treatment function. Wang and Ruan [6] introduced a constant treatment in an SIR model as follows:

(1) $$\begin{array}{c} h\left(I\right)=\{\begin{array}{cc} r, & I>0\\ 0, & I=0, \end{array} \end{array}$$

which simulated a limited capacity for treatment. Further, Wang [7] considered the following piecewise linear treatment function:

(2) $$\begin{array}{c} h\left(I\right)=\{\begin{array}{cc} rI, & 0\leq I\leq I_{0}\\ rI_{0}, & I>I_{0}, \end{array} \end{array}$$

where *I*_0_ is the infective level at which the health care system reaches capacity; that is, treatment increases linearly with *I* before the capacity is reached and then takes its maximum value *rI*_0_. This seems more reasonable than the usual linear function. In [8], J. C. Eckalbar and W. L. Eckalbar constructed an SIR model with a quadratic treatment function as follows:

(3) $$\begin{array}{c} T\left(I\right)=\max\left\{rI-gI^{2},0\right\},\;r,g>0. \end{array}$$

Besides this, we know that the efficiency of treatment will be seriously affected if the infective individuals are delayed for treatment. In [9], Zhang and Liu used a continuous and differentiable saturated treatment function *h*(*I*) = *rI*/(1 + *kI*), where *r* > 0,  *k* ≥ 0, *r* stands for the cure rate, and the parameter *k* measures the extent of the infected being delayed for treatment. It can be seen that the treatment function *h*(*I*) approaches *rI* when *I* is small; however, *h*(*I*) approaches *r*/*k* when *I* is large. It is more realistic and has the convenience of being continuous and differential than the previous ones.

Although the dynamics of SIR or SIS epidemic models with the saturated incidence rate have been frequently used in many literatures [9–12], there are not many researches about the saturated treatment function even in the SEIR epidemic models.

Motivated by these points, to better understand their effects on the spreading of infectious diseases, in this paper, we will discuss the SEIR model with the saturated incidence rate and the saturated treatment function. We suppose that, in incubation period, the hosts who have been infected by viruses do not have the ability to infect other hosts and the recovered individuals and vaccinated-treated individuals have gained permanent immunity and can no longer be infected.

The paper is organized as follows. In Section 2, we explore the existence of disease-free equilibrium point, endemic equilibrium point, and the existence of backward bifurcation and investigate the effect of the limited medical resources and their supply efficiency. In Section 3, we analyze the local asymptotic stability of the disease-free equilibrium and the endemic equilibrium. In Section 4, we analyze the global asymptotic stability of the disease-free and endemic equilibria. In Section 5, the paper ends with some numerical simulations to support and complement the theoretical findings.

## 2. The Model and the Existence of Equilibria and Bifurcation Analysis

In [12], a simple example is the classical SIR epidemic model with limited medical resources which carefully investigated the dynamics of the following SIR model:

(4) $$\begin{array}{c} S^{\prime }\left(t\right)=\Lambda -\frac{\beta SI}{1+kI}-dS,\\ I^{\prime }\left(t\right)=\frac{\beta SI}{1+kI}-\left(d+\gamma +\varepsilon \right)I-\frac{\alpha I}{\omega +I},\\ R^{\prime }\left(t\right)=\gamma I-dR+\frac{\alpha I}{\omega +I}. \end{array}$$

In [2], another simple example is the classical SEIR epidemic model with bilinear incidence rate which was studied as follows:

(5) $$\begin{array}{c} S^{\prime }\left(t\right)=A-\beta SI-\mu S,\\ E^{\prime }\left(t\right)=\beta SI-\left(\mu +\varepsilon \right)E,\\ I^{\prime }\left(t\right)=\varepsilon E-\left(\mu +r+d\right)I-\frac{cI}{b+I},\\ R^{\prime }\left(t\right)=rI-\mu R+\frac{cI}{b+I}. \end{array}$$

Based on the above motivations, in this paper, we further explore the SEIR epidemic model with saturated incidence rate *βSI*/(1 + *αI*) and a continually differentiable treatment function *h*(*I*) = *rI*/(1 + *kI*) (see, e.g., [9]) to characterize the saturation phenomenon of the limited medical resources. The model can be described by the following system of equations:

(6) $$\begin{array}{c} S^{\prime }\left(t\right)=A-\frac{\beta SI}{1+\alpha I}-dS,\\ E^{\prime }\left(t\right)=\frac{\beta SI}{1+\alpha I}-\left(d+\varepsilon \right)E,\\ I^{\prime }\left(t\right)=\varepsilon E-\left(d+\mu +\upsilon \right)I-\frac{rI}{1+kI},\\ R^{\prime }\left(t\right)=\upsilon I-dR+\frac{rI}{1+kI}, \end{array}$$

where *S*(*t*), *E*(*t*), *I*(*t*), and *R*(*t*) ≥ 0 and *S*(*t*), *E*(*t*), *I*(*t*), and *R*(*t*) denote the numbers of susceptible, exposed but not yet infectious, infective, and recovered individuals at time *t*, respectively. *A* is the recruitment rate of the population, *α* is the saturation factor that measures the inhibitory effect, *β* is the transmission or contact rate, *d* is the natural death rate of the population, *ε* is the rate of transformation from incubation period individuals to infective individuals, *μ* is the disease-related mortality, *υ* is the natural recovery rate of the infective individuals, *r* is the maximal medical resources supplied per unit time, and *k* is the saturation factor that measures the effect of the infected being delayed for treatment. *β*, *d*, *ε*, *μ*, *υ*, and *r* are all positive and *α* and *k* are nonnegative.

Since the first three equations in (6) are independent of the variable *R*, it suffices to consider the following reduced model:

(7) $$\begin{array}{c} S^{\prime }\left(t\right)=A-\frac{\beta SI}{1+\alpha I}-dS,\\ E^{\prime }\left(t\right)=\frac{\beta SI}{1+\alpha I}-\left(d+\varepsilon \right)E,\\ I^{\prime }\left(t\right)=\varepsilon E-\left(d+\mu +\upsilon \right)I-\frac{rI}{1+kI}. \end{array}$$

It follows from system (7) that

(8) (S+E+I)′=A−d(S+E+I)−(μ+υ)I−rI1+kI≤A−d(S+E+I).

Then limsup⁡_*t*→*∞*_(*S* + *E* + *I*) ≤ *A*/*d*. Thus the feasible region for system (7) is

(9) $$\begin{array}{c} \Omega =\left\{\left(S,E,I\right)\;|\;S+E+I\leq \frac{A}{d},S>0,E\geq 0,I\geq 0\right\}. \end{array}$$

It is easy to verify that the region *Ω* is positively invariant with respect to system (7).

Denote

(10) $$\begin{array}{c} R_{0}=\frac{\beta A\varepsilon }{d\left(d+\varepsilon \right)\left(d+\mu +\upsilon +r\right)}. \end{array}$$

The system (7) always has a disease-free equilibrium *E*_0_ = (*A*/*d*, 0,0). Next, we will find the conditions of the existence of endemic equilibrium.

An endemic equilibrium always satisfies

(11) $$\begin{array}{c} A-\frac{\beta SI}{1+\alpha I}-dS=0,\\ \frac{\beta SI}{1+\alpha I}-\left(d+\varepsilon \right)E=0,\\ \varepsilon E-\left(d+\mu +\upsilon \right)I-\frac{rI}{1+kI}=0. \end{array}$$

By some simple calculation, we have S* = *A*(1 + αI*)/(βI* + *d*(1 + αI*)), E* = *A* βI*/((*d* + *ε*)(*β* + *αd*)I* + *d*(*d* + *ε*)), and I* is the positive solution of the following equation:

(12) $$\begin{array}{c} aI^{*2}+bI^{*}+c=0, \end{array}$$

with

(13) $$\begin{array}{c} a=k\left(d+\varepsilon \right)\left(d+\mu +\upsilon \right)\left(\beta +\alpha d\right),\\ b=\left(d+\varepsilon \right)\left[\left(d+\mu +\upsilon \right)\left(\beta +\alpha d+kd\right)+r\left(\beta +\alpha d\right)\right]-\beta A\varepsilon k,\\ c=d\left(d+\varepsilon \right)\left(d+\mu +\upsilon +r\right)\left(1-R_{0}\right). \end{array}$$

For the endemic equilibrium to exist, the solutions of (12) must be real and positive.

We note

(14) $$\begin{array}{c} a\geq 0;\;c<0⟺R_{0}>1;\;c\geq 0⟺R_{0}\leq 1. \end{array}$$

Equation (12) is a quadratic equation with respect to I* since *a* > 0. Let the discriminant of (12) be Δ, so that Δ = *b* ^2^ − 4*ac*. Solving for Δ = 0 in terms of *R*_0_, we get *R*_0_ = *R*_0_^*c*^, where

(15) $$\begin{array}{c} R_{0}^{c}=1-\frac{b^{2}}{4ad\left(d+\varepsilon \right)\left(d+\mu +\upsilon +r\right)}. \end{array}$$

We can clearly note the following equivalent relations:

(16) $$\begin{array}{c} \Delta <0⟺R_{0}<R_{0}^{c},\;\;\Delta =0⟺R_{0}=R_{0}^{c},\\ \Delta >0⟺R_{0}>R_{0}^{c}. \end{array}$$

We thus have the following results on existence of the endemic equilibrium.

**Theorem 1 .**

The following results hold.

(*H*1) Let *k* = 0. Equation (12) is a linear equation with a unique solution *I* = −*c*/*b*. Then the system (7) has a unique endemic equilibrium when *R*_0_ > 1 and has no endemic equilibrium when *R*_0_ ≤ 1.
(*H*2) Let *k* > 0;
  1. system (7) has a unique endemic equilibrium whenever *R*_0_ > 1;
  2. system (7) has a unique endemic equilibrium whenever *R*_0_ = 1 and *b* < 0;
  3. system (7) has a unique endemic equilibrium of multiplicity 2 when *R*_0_ = *R*_0_^*c*^ and *b* < 0;
  4. system (7) has two endemic equilibria *E*_1_(*S*_1_, *E*_1_, *I*_1_) and *E*_2_(*S*_2_, *E*_2_, *I*_2_), when *R*_0_^*c*^ < *R*_0_ < 1 and *b* < 0, where $I_{1}=\left(-b+\sqrt{b^{2}-4ac}\right)/2a$ and $I_{2}=\left(-b-\sqrt{b^{2}-4ac}\right)/2a$;
  5. system (7) has no endemic equilibria whenever *R*_0_ < *R*_0_^*c*^ and *b* < 0 or whenever *R*_0_ ≤ 1 and *b* > 0.

From Theorem 1, we know that if *k* = 0, there is a unique endemic equilibrium when *R*_0_ > 1 which approaches zero as *R*_0_ → 1_+_ and there cannot be an endemic equilibrium if *R*_0_ < 1. In this case it is impossible to have a backward bifurcation at *R*_0_ = 1. However, if *k* > 0,  *b* < 0, system (7) has a unique endemic equilibrium when *R*_0_ > 1 and has two different endemic equilibria when *R*_0_^*c*^ < *R*_0_ < 1, and (7) has no endemic equilibrium when 0 < *R*_0_ < *R*_0_^*c*^. Hence, (7) has a backward bifurcation at *R*_0_ = 1 from the disease-free equilibrium to two endemic equilibria. To conclude, we have the following theorem.

**Theorem 2 .**

System (7) has a backward bifurcation at *R*_0_ = 1 if and only if *k* > 0,  *b* < 0.

**Proof**

For sufficiency, let us consider the graph of *y* = *f*(*x*) = *ax* ^2^ + *bx* + *c*. It passes through the origan since *c* = 0 when *R*_0_ = 1. Further, if *b* < 0, we have that *f*(*x*) = 0 has a positive root. Now we increase *c* to *c* > 0; the fact that *f*(*x*) is a continuous function of *c* guarantees that there will be some open interval of *c*, say (0, *ε*), on which *f*(*x*) = 0 has two positive real roots. In other words, we have shown that it is possible that there exist two endemic equilibria when *R*_0_ < 1.

The necessary is obvious, since, if *b* ≥ 0, (12) has no positive real root when *R*_0_ < 1, thereby completing the proof.

Under the condition of Theorem 2, we give an explicit criterion of *k* in terms of the parameters *β*, *a*, *d*, *μ*, *υ*, *r* for the existence of a backward bifurcation at *R*_0_ = 1.

**Corollary 3 .**

When *k* > *k*_0_, then system (7) has a backward bifurcation at *R*_0_ = 1, where *k*_0_ = (*β* + *ad*)(*d* + *μ* + *υ* + *r*)/*dr*.

**Proof**

When *R*_0_ = 1⇔*c* = 0,

(17) $$\begin{array}{c} \beta A\varepsilon =d\left(d+\varepsilon \right)\left(d+\mu +\upsilon +r\right). \end{array}$$

The condition *b* < 0 is equivalent to

(18) $$\begin{array}{c} \left(d+\varepsilon \right)\left[\left(d+\mu +\upsilon \right)\left(\beta +ad+kd\right)+r\left(\beta +ad\right)\right]<\beta A\varepsilon k. \end{array}$$

From (17) and (18), we get

(19) (d+ε)[(d+μ+υ)(β+ad+kd)+r(β+ad)]  <kd(d+ε)(d+μ+υ+r),

which reduces to

(20) $$\begin{array}{c} k>\frac{\left(\beta +ad\right)\left(d+\mu +\upsilon +r\right)}{dr}≜k_{0}. \end{array}$$

**Figure 1:**
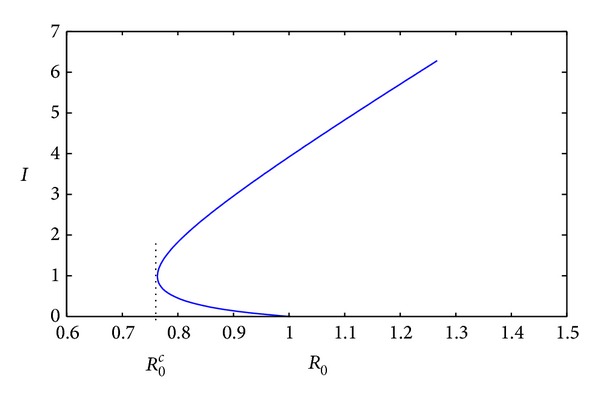
The figure of infective sizes at equilibria versus *R*_0_ when *β* = 0.05, *ε* = 1.2, *d* = 0.2, *μ* = 0.2, *υ* = 0.4, *r* = 1.5, *α* = 0.1, and *k* = 2, where *k* is big enough to lead a backward bifurcation with two endemic equilibria when *R*_0_^*c*^ < *R*_0_ < 1.

So a backward bifurcation occurs at *R*_0_ = 1 if and only if (20) is satisfied. Further, from this we can point out that when the effect of the infected being delayed for treatment becomes stronger than some level, the backward bifurcation will take place. Thus the effect of the infected being delayed for treatment, say *k*, is one of the factors which lead to the backward bifurcation (see Figure 1).

In order to verify the bifurcation curve (the graph of *I* as a function of *R*_0_) in Figure 1, we think of *r* as a variable with the other parameters as constant. Through implicit differentiation of (12) with respect to *r*, we get

(21) $$\begin{array}{c} \left(2aI+b\right)\frac{\text{d}I}{\text{d}r}=-d\left(d+\varepsilon \right)-\left(d+\varepsilon \right)\left(\beta +ad\right)I<0. \end{array}$$

From (21) we know the sign of d*I*/d*r* is opposite to that of 2*aI* + *b*. And from the definition of *R*_0_ we know that *R*_0_ decreases when *r* increase. It implies that the bifurcation curve has positive slope at equilibrium values with 2*aI* + *b* > 0 and negative slope at equilibrium values with 2*aI* + *b* < 0. If there is no backward bifurcation at *R*_0_ = 1, then the unique endemic equilibrium for *R*_0_ > 1 satisfies

(22) $$\begin{array}{c} 2aI+b=\sqrt{b^{2}-4ac}>0, \end{array}$$

and the bifurcation curve has positive slope at all points where *I* > 0. If there is a backward bifurcation at *R*_0_ = 1, then there is an interval (*R*_0_^*c*^, 1) on which there are two endemic equilibria given by

(23) $$\begin{array}{c} 2aI+b=\pm \sqrt{b^{2}-4ac}. \end{array}$$

The bifurcation curve has negative slope at the smaller one and positive slope at the larger one. Thus the bifurcation curve is shown in Figure 1. Under the conditions of Theorem 1, if a backward bifurcation takes place, we can see from Figure 1 there is a critical value *R*_0_^*c*^ at the turning point. In this case, the disease will not die out when *R*_0_ < 1. However, the disease will die out when *R*_0_ < *R*_0_^*c*^. Therefore, the critical value *R*_0_^*c*^ can be taken as a new threshold for the control of the disease.

## 3. The Local Stability Analysis of Equilibria

In this section, we will examine the local stability of the equilibria by analyzing the eigenvalues of the Jacobian matrices of (7) at the equilibria and using Routh-Hurwitz criterion.

**Theorem 4 .**

The disease-free equilibrium *E*_0_ is locally asymptotically stable when *R*_0_ < 1 and is unstable when *R*_0_ > 1.

**Proof**

The Jacobian matrix of (7) at *E*_0_ is

(24) $$\begin{array}{c} J\left(E_{0}\right)=\left(\begin{array}{ccc} -d & 0 & -\frac{\beta A}{d}\\ 0 & -\left(d+\varepsilon \right) & \frac{\beta A}{d}\\ 0 & \varepsilon & -\left(d+\mu +\upsilon +r\right) \end{array}\right). \end{array}$$

The characteristic equation of system (7) at *E*_0_ is of the following form:

(25) $$\begin{array}{c} \left(\lambda +d\right)\left(\lambda^{2}+P\lambda +Q\right)=0, \end{array}$$

where *P* = 2*d* + *μ* + *υ* + *r* + *ε*, *Q* = (*d* + *ε*)(*d* + *μ* + *υ* + *r*) − (*βAε*/*d*).

Clearly, *λ* = −*d* is always a root of (25). All other roots of (25) are determined by the following equation:

(26) $$\begin{array}{c} \lambda^{2}+P\lambda +Q=0, \end{array}$$

which has negative roots, if and only if (*d* + *ε*)(*d* + *μ* + *υ* + *r*) − (*βAε*/*d*) > 0. This condition is equivalent to *R*_0_. So the disease-free equilibrium *E*_0_ is locally asymptotically stable when *R*_0_ < 1 and is unstable when *R*_0_ > 1.

**Theorem 5 .**

When *R*_0_ > 1 and 0 ≤ *k* < *k*_1_, the unique endemic equilibrium *E*_∗_(S*, E*, I*) is locally asymptotically stable, where *k*_1_ = *α*(*d* + *μ* + *υ* + *r*)/*r*.

**Proof**

The Jacobian matrix of (7) at *E*_∗_ is

(27) J(E∗)=(−d−βI∗1+αI∗0−βS∗(1+αI∗)2βI∗1+αI∗−(d+ε)βS∗(1+αI∗)20ε−(d+μ+υ)−r(1+kI∗)2).

The characteristic equation is

(28) |λ+d+βI∗1+αI∗0βS∗(1+αI∗)2−βI∗1+αI∗λ+(d+ε)−βS∗(1+αI∗)20−ελ+(d+μ+υ)+r(1+kI∗)2|  =0,

that is,

(29) $$\begin{array}{c} \lambda^{3}+a_{1}\lambda^{2}+a_{2}\lambda +a_{3}=0, \end{array}$$

where

(30) a1=d+βI∗1+αI∗+2d+ε+μ+υ+r(1+kI∗)2>0,a2=(d+βI∗1+αI∗)[2d+ε+μ+υ+r(1+kI∗)2]+(d+ε)[d+μ+υ+r(1+kI∗)2]−βS∗ε(1+αI∗)2,a3=(d+βI∗1+αI∗)×[(d+ε)(d+μ+υ+r(1+kI∗)2)−βS∗ε(1+αI∗)2]+βI∗1+αI∗βS∗ε(1+αI∗)2.

From the second and third equations of (11), we have

(31) $$\begin{array}{c} \frac{\beta S^{*}\varepsilon }{1+\alpha I^{*}}=\left(d+\varepsilon \right)\left(d+\mu +\upsilon +\frac{r}{1+kI^{*}}\right). \end{array}$$

Let *M* = (*d* + *ε*)(*d* + *μ* + *υ* + (*r*/(1 + kI*)^2^)) − (βS*ε/(1 + αI*)^2^). From (31), we get

(32) M=I∗(d+ε)(1+αI∗)(1+kI∗)2×[α(d+μ+υ)(1+kI∗)2+r(α−k)],

which is positive if and only if *α*(*d* + *μ* + *υ*)(1 + kI*)^2^ + *rα* > *rk*.

In fact, we have

(33) α(d+μ+υ)(1+kI∗)2+rα>α(d+μ+υ)+rα=α(d+μ+υ+r).

So *M* is positive if

(34) $$\begin{array}{c} \alpha \left(d+\mu +\upsilon +r\right)>rk, \end{array}$$

or

(35) $$\begin{array}{c} k<\frac{\alpha \left(d+\mu +\upsilon +r\right)}{r}≜k_{1}. \end{array}$$

It follows from *k* < *k*_1_ that *a*_3_ > 0.

By a direct calculation, we have that *H*_1_ = *a*_1_ > 0, *H*_2_ = *a*_1_ *a*_2_ − *a*_3_ > 0, and *H*_3_ = *a*_3_(*a*_1_ *a*_2_ − *a*_3_) > 0 under the condition *k* < *k*_1_. Then by Routh-Hurwitz criterion, it follows that the endemic equilibrium *E*_∗_ is locally asymptotically stable. This completes the proof.

## 4. The Global Stability Analysis of Equilibria

In this section, we analyze the global stability of the disease-free and endemic steady states. Firstly, we consider the global stability of the disease-free equilibrium.

Define

(36) $$\begin{array}{c} R_{0}^{*}=\frac{\varepsilon \beta A}{d\left(d+\varepsilon \right)\left(d+\mu +\upsilon +\left(r/\left(1+\alpha \left(A/d\right)\right)\right)\right)}. \end{array}$$

**Theorem 6 .**

If *R*_0_* < 1, then the disease-free equilibrium *E*_0_ is globally asymptotically stable.

**Proof**

If *R*_0_* < 1, then *R*_0_ < 1. From the first equation of (6), we have *dS*/*dt* ≤ *A* − *dS*. A solution of the equation *dy*/*dt* = *A* − *dy* is a maximal solution of *S*(*t*). Note that *y* → *A*/*d* as *t* → *∞*. By the comparison theorem, we get *S*(*t*) ≤ *A*/*d*, and from the set *Ω* = {(*S*, *E*, *I*)∣*S* + *E* + *I* ≤ *A*/*d*, *S* > 0, *E* ≥ 0, *I* ≥ 0} we have *I*(*t*) ≤ *A*/*d*.

Consider the following Lyapunov function:

(37) $$\begin{array}{c} L=\varepsilon E+\left(d+\varepsilon \right)I. \end{array}$$

From *R*_0_* < 1, we have *εβ*(*A*/*d*) − (*d* + *ε*)(*d* + *μ* + *υ* + (*r*/(1 + *α*(*A*/*d*)))) < 0. Thus,

(38) L′=[εβS1+αI−(d+ε)(d+μ+υ+r1+αI)]I≤[εβS−(d+ε)(d+μ+υ+r1+αI)]I≤[εβAd−(d+ε)(d+μ+υ+r1+α(A/d))]I≤0,

and *L*′ = 0 if and only if *I* = 0. The largest compact invariant set in {(*S*, *E*, *I*) ∈ *Ω*, *L*′ = 0} is the singleton *E*_0_. Therefore, by Lasalle-Lyapunov theorem, every solution that starts in *Ω* approaches *E*_0_ as *t* → *∞*. This completes the proof.

In the following, we will discuss the global stability of the endemic equilibrium when *R*_0_ > 1, *k* < *k*_1_ using the second additive compound matrix. Here we will shortly describe the general method in which the global stability analysis for the endemic equilibrium will be performed through the approach due to Li and Muldowney [13]. Consider the autonomous dynamical system

(39) $$\begin{array}{c} x^{\prime }=f\left(x\right), \end{array}$$

where *f* : *D* → *R* ^*n*^, *D* ⊂ *R* ^*n*^ is open set and is simply connected, and *x* ∈ *D*, *x* ↦ *f*(*x*) ∈ *R* ^*n*^, *f*(*x*) ∈ *C* ^1^(*D*).

Let x* be an equilibrium of (39). We recall that x* is said to be globally stable in *D* if it is locally stable and all trajectories in *D* converge to x*. Assume that the following hypotheses hold.

(*H*1) There exists a compact absorbing set *K* ⊂ *D*.
(*H*2) Equation (39) has a unique equilibrium x* in *D*.

The basic idea of this method is that if the equilibrium x* is locally stable, then the stability is assured provided that (*H*1) and (*H*2) hold and no nonconstant periodic solution of (39) exists. Therefore, sufficient conditions on *f* capable of precluding the existence of such solutions have to be detected.

Li and Muldowney showed that if (*H*1) and (*H*2) hold and (39) satisfies a Bendixson criterion that is robust under *C* ^1^ local *ϵ*-perturbations of *f* at all nonequilibrium nonwandering points for (39), then x* is globally stable and robust under *C* ^1^ local *ε*-perturbation. Let *P*(*x*) be a $\begin{array}{c} \left(\begin{array}{c} n\\ 2 \end{array}\right)\times \left(\begin{array}{c} n\\ 2 \end{array}\right) \end{array}$ matrix-valued function, that is, *C* ^1^, on *D* and consider

(40) $$\begin{array}{c} B=P_{f}P^{-1}+P\frac{\partial f^{\left[2\right]}}{\partial x}P^{-1}, \end{array}$$

where the matrix *P*_*f*_ is

(41) $$\begin{array}{c} \frac{\partial P_{ij}^{*}}{\partial x}f={\frac{\text{d}P_{ij}}{\text{d}t}|}_{\left(39\right)}, \end{array}$$

and the matrix *J* ^[2]^ is the second additive compound matrix of the Jacobian matrix *J*, that is, *J*(*x*) = *Df*(*x*). Generally speaking, for an *n* × *n* matrix *J* = (*J*_*ij*_), *J* ^[2]^ is a $\begin{array}{c} \left(\begin{array}{c} n\\ 2 \end{array}\right)\times \left(\begin{array}{c} n\\ 2 \end{array}\right) \end{array}$ matrix and in the special case *n* = 3 one has

(42) $$\begin{array}{c} J^{\left[2\right]}=\left(\begin{array}{ccc} J_{11}+J_{22} & J_{23} & -J_{13}\\ J_{32} & J_{11}+J_{33} & J_{12}\\ -J_{31} & J_{21} & J_{22}+J_{33} \end{array}\right). \end{array}$$

Consider the $\text{Lozinski}\overset{˘}{\text{l}}$ measure *μ* of B with respect to a vector norm |·| in *R* ^*N*^, $\begin{array}{c} N=\left(\begin{array}{c} n\\ 2 \end{array}\right) \end{array}$ (see [14]):

(43) $$\begin{array}{c} \mu \left(B\right)=\underset{h\to 0^{+}}{\lim}\frac{||I+hB||-1}{h}. \end{array}$$

It is proved in [13] that if (*H*_1_) and (*H*_2_) hold, condition

(44) $$\begin{array}{c} q=\underset{t\to \infty }{\mathrm{limsup}}\underset{x_{0}\in K}{\sup}\frac{1}{t}\overset{t}{\underset{0}{\int }}\mu \left(B\left(x\left(s,x_{0}\right)\right)\right)\text{d}s<0 \end{array}$$

guarantees that there are no orbits giving rise to a simple closed rectifiable curve in *D* which is invariant for (39), that is, periodic orbits, homoclinic orbits, and heteroclinic cycles. In particular, condition (44) is proved to be a robust Bendixson criterion for (39). Besides, it is remarked that, under assumptions (H1) and (H2), condition (44) also implies the local stability of x*.

The analysis of the global stability of the endemic equilibrium may be usefully approached by means of the Poincare-Bendixson trichotomy. If the endemic equilibrium is globally asymptotically stable, then the disease will permanently be present in the population in case of infinitesimal initial prevalence. Here we will provide an analytical proof of global stability of *E*_∗_ by giving sufficient conditions. Global stability analysis for the endemic equilibrium will be performed through the approach due to Li and Muldowney. The instability of *E*_0_ implies the uniform persistence; that is, there exists a constant *a* > 0 such that any solution (*S*(*t*), *E*(*t*), *I*(*t*)) with (*S*(0), *E*(0), *I*(0)) in the orbit of the system satisfies

(45) $$\begin{array}{c} \min\left\{\underset{t\to \infty }{\mathrm{liminf}}S\left(t\right),\underset{t\to \infty }{\mathrm{liminf}}E\left(t\right),\underset{t\to \infty }{\mathrm{liminf}}I\left(t\right)\right\}>a. \end{array}$$

**Lemma 7 (see [13]).**

Assume that conditions (*H*_1_) and (*H*_2_) hold; then x* is globally asymptotically stable in *D* provided that a function *P*(*x*) and a $\text{Lozinski}\overset{˘}{\text{l}}$ measure *μ* exist such that condition (44) is satisfied.

**Theorem 8 .**

Under the condition *R*_0_ > 1, 0 ≤ *k* < *k*_1_, *d* > *r*, the endemic equilibrium *E*_∗_ of the system (7) is globally asymptotically stable.

**Proof**

The Jacobian matrix of system (7) is

(46) $$\begin{array}{c} J=\left(\begin{array}{ccc} -d-\frac{\beta I}{1+\alpha I} & 0 & -\frac{\beta S}{{\left(1+\alpha I\right)}^{2}}\\ \frac{\beta I}{1+\alpha I} & -\left(d+\varepsilon \right) & \frac{\beta S}{{\left(1+\alpha I\right)}^{2}}\\ 0 & \varepsilon & -\left(d+\mu +\upsilon \right)-\frac{r}{{\left(1+kI\right)}^{2}} \end{array}\right), \end{array}$$

and its second additive compound matrix is

(47) $$\begin{array}{c} J^{\left[2\right]}=\left(\begin{array}{ccc} -\left(2d+\varepsilon \right)-\frac{\beta I}{1+\alpha I} & \frac{\beta S}{{\left(1+\alpha I\right)}^{2}} & \frac{\beta S}{{\left(1+\alpha I\right)}^{2}}\\ \varepsilon & -\frac{\beta I}{1+\alpha I}-\left(2d+\mu +\upsilon \right)-\frac{r}{{\left(1+kI\right)}^{2}} & 0\\ 0 & \frac{\beta I}{1+\alpha I} & -\left(2d+\varepsilon +\mu +\upsilon \right)-\frac{r}{{\left(1+kI\right)}^{2}} \end{array}\right). \end{array}$$

Choose the function *P* = *P*(*S*, *E*, *I*) = diag⁡(1, *E*/*I*, *E*/*I*); then

(48) $$\begin{array}{c} P_{f}=\mathrm{diag}\left(\begin{array}{c} 0,\frac{E^{\prime }I-I^{\prime }E}{I^{2}},\frac{E^{\prime }I-I^{\prime }E}{I^{2}} \end{array}\right). \end{array}$$

It follows that

(49) $$\begin{array}{c} P_{f}P^{-1}=\mathrm{diag}\left(\begin{array}{c} 0,\frac{E^{\prime }}{E}-\frac{I^{\prime }}{I},\frac{E^{\prime }}{E}-\frac{I^{\prime }}{I} \end{array}\right),\\ PJ^{\left[2\right]}P^{-1}=\left(\begin{array}{ccc} -\left(2d+\varepsilon \right)-\frac{\beta I}{1+\alpha I} & \frac{\beta SI}{{\left(1+\alpha I\right)}^{2}E} & \frac{\beta SI}{{\left(1+\alpha I\right)}^{2}E}\\ \frac{\varepsilon E}{I} & -\frac{\beta I}{1+\alpha I}-\left(2d+\mu +\upsilon \right)-\frac{r}{{\left(1+kI\right)}^{2}} & 0\\ 0 & \frac{\beta I}{1+\alpha I} & -\left(2d+\varepsilon +\mu +\upsilon \right)-\frac{r}{{\left(1+kI\right)}^{2}} \end{array}\right). \end{array}$$

The matrix *B* = *P*_*f*_ *P* ^−1^ + *PJ* ^[2]^ *P* ^−1^ can be written in matrix form

(50) $$\begin{array}{c} B=\left(\begin{array}{cc} B_{11} & B_{12}\\ B_{21} & B_{22} \end{array}\right), \end{array}$$

where

(51) $$\begin{array}{c} B_{11}=-\left(2d+\varepsilon \right)-\frac{\beta I}{1+\alpha I},\;\;B_{12}=\left(\frac{\beta SI}{{\left(1+\alpha I\right)}^{2}E},\frac{\beta SI}{{\left(1+\alpha I\right)}^{2}E}\right),\;\;B_{21}={\left(\frac{\varepsilon E}{I},0\right)}^{T},\\ B_{22}=\left(\begin{array}{cc} -\frac{\beta I}{1+\alpha I}-\frac{r}{{\left(1+kI\right)}^{2}}+\frac{E^{\prime }}{E}-\frac{I^{\prime }}{I}-\left(2d+\mu +\upsilon \right) & 0\\ \frac{\beta I}{1+\alpha I} & -\frac{r}{{\left(1+kI\right)}^{2}}+\frac{E^{\prime }}{E}-\frac{I^{\prime }}{I}-\left(2d+\varepsilon +\mu +\upsilon \right) \end{array}\right). \end{array}$$

Let (*u*, *v*, *ω*) be a vector in *R* ^3^; its norm ||·|| is defined as

(52) $$\begin{array}{c} ||\left(u,v,\omega \right)||=\max\left\{\left|u\right|,\left|v\right|+\left|\omega \right|\right\}. \end{array}$$

Let *μ*(*B*) be the $\text{Lozinski}\overset{˘}{\text{l}}$ measure with respect to this norm. We choose

(53) $$\begin{array}{c} \mu \left(B\right)\leq \sup\left\{g_{1},g_{2}\right\}, \end{array}$$

where *g*_1_ = *μ*_1_(*B*_11_)+|*B*_12_|, *g*_2_ = *μ*_1_(*B*_22_)+|*B*_21_|, |*B*_12_|, |*B*_21_| are matrix norms with respect to *l*_1_ vector norm, and *μ*_1_ denotes the $\text{Lozinski}\overset{˘}{\text{l}}$ measure with respect to this *l*_1_ norm; then

(54) $$\begin{array}{c} \mu_{1}\left(B_{11}\right)=-\frac{\beta I}{1+\alpha I}-\left(2d+\varepsilon \right),\\ \left|B_{12}\right|=\frac{\beta SI}{{\left(1+\alpha I\right)}^{2}E},\;\;\left|B_{21}\right|=\frac{\varepsilon E}{I}. \end{array}$$

Next calculating *μ*_1_(*B*_22_), taking the nondiagonal elements of each column of *B*_22_ in absolute value, and then adding to the corresponding columns of the diagonal elements, we get

(55) $$\begin{array}{c} B_{22}^{\prime }=\left(\begin{array}{cc} -\frac{r}{{\left(1+kI\right)}^{2}}+\frac{E^{\prime }}{E}-\frac{I^{\prime }}{I}-\left(2d+\mu +\upsilon \right) & 0\\ \frac{\beta I}{1+\alpha I} & -\frac{r}{{\left(1+kI\right)}^{2}}+\frac{E^{\prime }}{E}-\frac{I^{\prime }}{I}-\left(2d+\varepsilon +\mu +\upsilon \right) \end{array}\right). \end{array}$$

Take a maximum of two diagonal elements of *B*_22_′; we have

(56) μ1(B22) =max⁡⁡{−r(1+kI)2+E′E−I′I−(2d+μ+υ),     −r(1+kI)2+E′E−I′I−(2d+ε+μ+υ)} =−r(1+kI)2+E′E−I′I−(2d+μ+υ).

Therefore, we have

(57) g1=μ1(B11)+|B12|=βSI(1+αI)2E−βI1+αI−(2d+ε),g2=μ1(B22)+|B21|=εEI−r(1+kI)2+E′E−I′I−(2d+μ+υ).

From (6), we get

(58) $$\begin{array}{c} \frac{E^{\prime }}{E}=\frac{\beta SI}{\left(1+\alpha I\right)E}-\left(d+\varepsilon \right),\\ \frac{I^{\prime }}{I}=\frac{\varepsilon E}{I}-\frac{r}{1+kI}-\left(d+\mu +\upsilon \right). \end{array}$$

Then, we have

(59) g1=βSI(1+αI)2E−βI1+αI−(2d+ε)≤βSI(1+αI)E−(2d+ε)=E′E−d,

(60) g2=βSI(1+αI)E−(2d+ε)+r1+kI−r(1+kI)2=E′E−d+r1+kI−r(1+kI)2≤E′E−(d−r).

Furthermore, we obtain

(61) μ(B)≤sup⁡⁡{g1,g2}≤{E′E−d,E′E−(d−r)}≤E′E−(d−r).

By integrating both sides at the same time, we obtain

(62) $$\begin{array}{c} \frac{1}{t}\overset{t}{\underset{0}{\int }}\mu \left(B\right)\text{d}s\leq \frac{1}{t}\ln\frac{E\left(t\right)}{E\left(0\right)}-\left(d-r\right),\\ \underset{t\to \infty }{\mathrm{limsup}}\sup\frac{1}{t}\overset{t}{\underset{0}{\int }}\mu \left(B\right)\text{d}s<-\left(d-r\right)<0. \end{array}$$

The proof is completed by Lemma 7.

## 5. Numerical Simulations

To demonstrate the theoretical results obtained in this paper, we will give some numerical simulations. We consider the hypothetical set of parameter values as the following.

**Figure 2:**
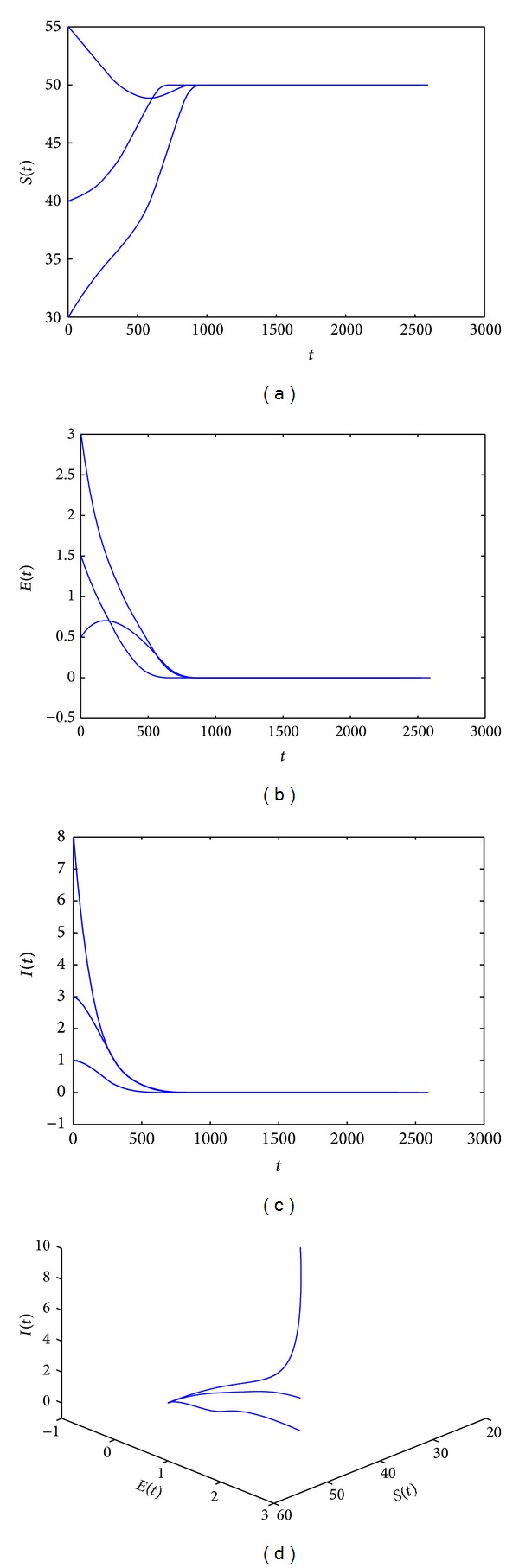
(a)–(d) show that system (7) has only one disease-free equilibrium *E*_0_(50,0, 0) and it is locally asymptotically stable. In this case, *A* = 10, *β* = 0.05, *ε* = 1.2, *d* = 0.2, *μ* = 0.2, *υ* = 0.4, *r* = 2.5, *α* = 1.25, and *k* = 1.25.

**Figure 3:**
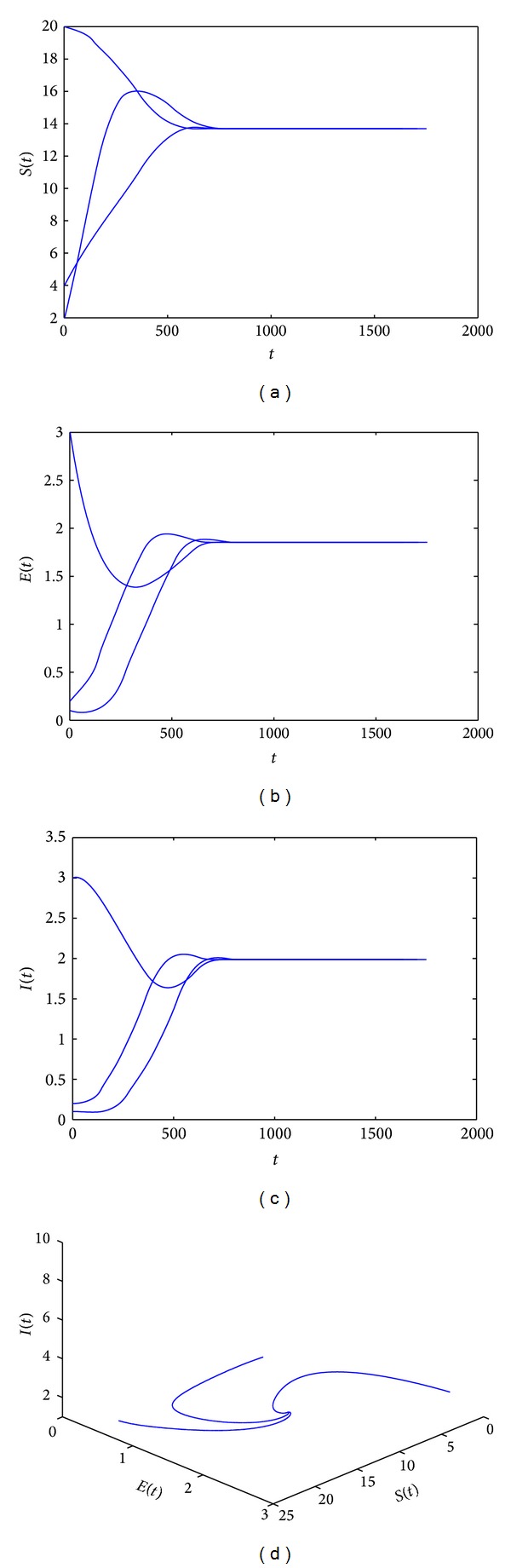
(a)–(d) show that system (7) has a disease-free equilibrium *E*_0_(50,0, 0), which is unstable, and an endemic equilibrium *E*_∗_(13.6954573045,1.8542772633,1.9865335214), which is locally asymptotically stable. In this case, *A* = 10, *β* = 0.3, *ε* = 1.2, *d* = 0.05, *μ* = 0.2, *υ* = 0.4, *r* = 0.1, *α* = 0.8, and *k* = 2.

**Figure 4:**
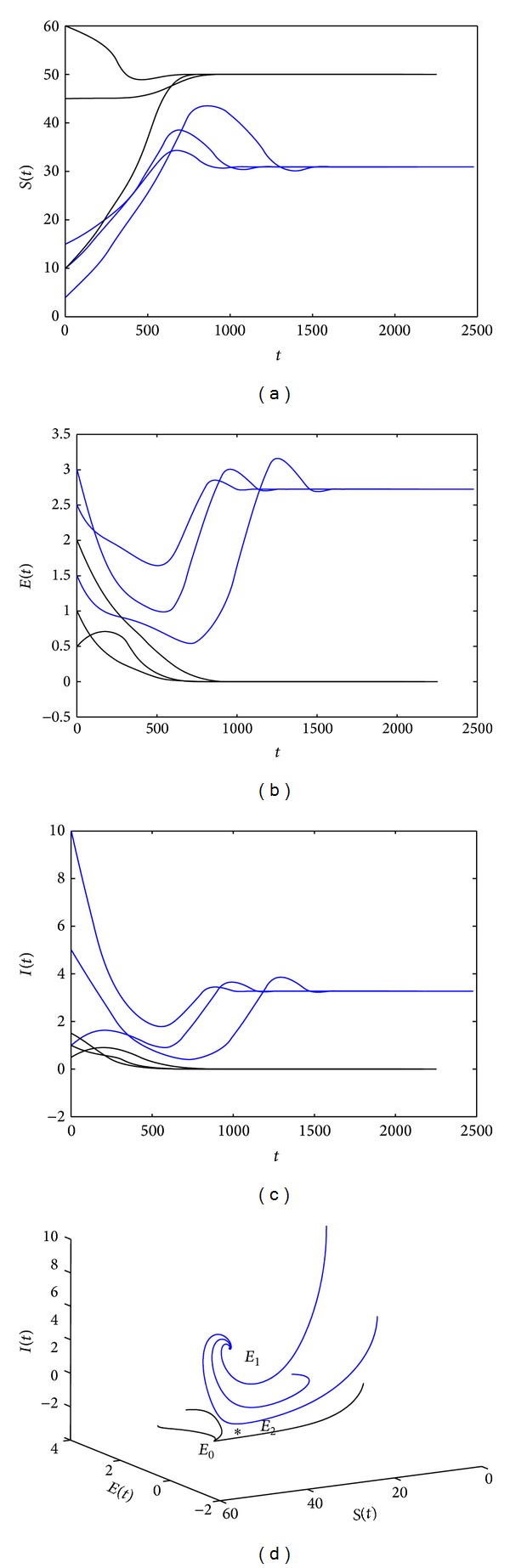
(a)–(d) show that system (7) has the bistable equilibria: a disease-free equilibrium *E*_0_(50,0, 0) and an endemic equilibrium *E*_1_(30.9324582801,2.7239504131,3.2726743508). And the other endemic equilibrium *E*_2_(48.9594277798,0.1486531743,0.08857440172) is unstable. In this case, *A* = 10, *β* = 0.05, *ε* = 1.2, *d* = 0.2, *μ* = 0.2, *υ* = 0.4, *r* = 1.5, *α* = 0.1, and *k* = 2.

1. *A* = 10, *β* = 0.05, *ε* = 1.2, *d* = 0.2, *μ* = 0.2, *υ* = 0.4, *r* = 2.5, *α* = 1.25, and *k* = 1.25. The condition of Theorem 4 is satisfied, that is, *R*_0_ = 0.6493506493 < 1. Then the system (7) has a disease-free equilibrium *E*_0_(50,0, 0) and it is globally asymptotically stable for this case (see Figures 2(a)–2(c) and 2(d)).
2. *A* = 10, *β* = 0.3, *ε* = 1.2, *d* = 0.5, *μ* = 0.2, *υ* = 0.4, *r* = 0.1, *α* = 0.8, and *k* = 2. Through calculation, we know *R*_0_ = 3.5294117647 > 1, *k* < *k*_1_ = 9.6, and *d* > *r*. According to Theorem 8, we know the endemic equilibrium *E*_∗_(13.6954573045,1.8542772633,1.9865335214) is globally asymptotically stable for this case (see Figures 3(a)–3(c) and 3(d)).
3. *A* = 10, *β* = 0.05, *ε* = 1.2, *d* = 0.2, *μ* = 0.2, *υ* = 0.4, *r* = 1.5, *α* = 0.1, and *k* = 2. Through calculation, we know *R*_0_ = 0.9316770185 < 1, and *R*_0_^*c*^ = 0.7634537608 < *R*_0_ < 1, *b* = −0.5266 < 0. From Theorem 1, we know system exists two endimic equilibria *E*_1_(30.9324582801,2.7239504131,3.2726743508) and *E*_2_(48.9594277798,0.1486531743,0.08857440172). The phase portrait related to this bistable situation is represented in Figure 4.

## 6. Conclusion

In this paper, we consider the SEIR epidemic model with saturated incidence and saturated treatment function to understand the effect of delayed treatment on the disease transmission. Generally speaking, in many epidemic models, the basic reproduction number, which is the key concept in epidemiology, can be decreased below unity to eradicate the disease. However, in our model, the basic reproduction number below unity is not enough to eradicate the disease. According to our analysis in this paper, we find that a backward bifurcation occurs when the capacity of the treatment is low (i.e., *k* > *k*_0_). If there is no delayed treatment (i.e., *k* = 0), system (7) only admits a forward bifurcation and the global dynamics are completely determined by the basic reproduction number *R*_0_. If there is delayed treatment (i.e., *k* > 0), then system (7) has much richer dynamics. For example, Corollary 3 suggests we must try our best to let *k* ≤ *k*_0_ to prevent the backward bifurcation. Through studying the bifurcation of our model, we suggest that, in order to eradicate the disease, we should raise the efficiency and enlarge the capacity of the treatment. That is to say, we should improve our medical technology and invest more medicines, beds, and so forth to give the patients timely treatment.

Lastly, a numerical simulation provided that when *R*_0_ < 1, the disease-free equilibrium is stable (see Figure 2), while *R*_0_ > 1, the disease-free equilibrium is unstable, and under the condition *k* < *k*_1_, the endemic equilibrium *E*_∗_ is globally asymptotically stable (see Figure 3). The stability of equilibria *E*_1_, *E*_2_ has not been studied, when *R*_0_^*c*^ < *R*_0_ < 1 and *b* < 0. It is worthwhile for us to study this case from the theorematic idea in the future work. Here we only illustrate that the equilibrium *E*_1_ is stable, while *E*_2_ is unstable by using the numerical simulation (see Figure 4).
